# Does clinical experience with dental traumatology impact 2D and 3D radiodiagnostic performance in paediatric dentists? An exploratory study

**DOI:** 10.1186/s12903-022-02281-4

**Published:** 2022-06-20

**Authors:** Gertrude Van Gorp, Marjan Lambrechts, Reinhilde Jacobs, Dominique Declerck

**Affiliations:** 1 Department of Oral Health Sciences and Department of Dentistry, Unit of Paediatric Dentistry and Special Dental Care, University Hospitals Leuven, KU Leuven, Kapucijnenvoer 7, PO box 7001, 3000 Leuven, Belgium; 2 OMFS IMPATH Research Group, Department of Imaging and Pathology, Faculty of Medicine, University of Leuven, Louvain, Belgium; 3 Oral and Maxillofacial Surgery, University Hospitals Leuven, Louvain, Sweden; 4 Department Dental Medicine, Karolinska Institutet, Stockholm, Sweden

**Keywords:** Paediatric dentists, Intraoral radiographs (2D), Cone beam CT (3D), Dental trauma, Diagnostic performance

## Abstract

**Background:**

The aim of this study is to evaluate the impact of experience with traumatic dental injuries (TDI) on paediatric dentists’ performance and self-assessed confidence when radiodiagnosing traumatic dental injuries (TDI) and to explore whether this is influenced by the imaging technique used (2D versus 3D).

**Materials and methods:**

Both 2D and 3D radiological images of young anterior permanent teeth having experienced dental trauma were assessed randomly by a panel of paediatric dentists using structured scoring sheets. The impact of level of experience with dental traumatology on radiological detection, identification and interpretation of lesions and on observer’s self-assessed confidence was evaluated. Findings were compared to benchmark data deriving from expert consensus of an experienced paediatric endodontologist and dentomaxillofacial radiologist. Results were analysed using generalized linear mixed modelling.

**Results:**

Overall, observers performed moderately to poor, irrespective of their level of TDI experience and imaging modality used. No proof could be yielded that paediatric dentists with high TDI experience performed better than those with low experience, for any of the outcomes and irrespective of the imaging modality used. When comparing the use of 3D images with 2D images, significantly higher sensitivities for the detection and correct identification of anomalies were observed in the low experienced group (*P* < 0.05). This was not the case regarding interpretation of the findings. Self-assessed confidence was significantly higher in more experienced dentists, both when using 2D and 3D images (*P* < 0.05).

**Conclusion:**

There was no proof that paediatric dentist’s higher experience with TDI is associated with better radiodiagnostic performance. Neither could it be proven that the use of Cone Beam Computed Tomography (CBCT) contributes to an improved interpretation of findings, for any experience level. More experienced dentists feel more confident, irrespective of the imaging modality used, but this does not correlate with improved performance. The overall poor performance in image interpretation highlights the importance of teaching and training in both dental radiology and dental traumatology.

## Background

Traumatic dental injuries (TDI) are quite common in children affecting an estimated 18% of 12-year-olds [1]. The most common types of traumatic dental injury occurring in children between the age of 7 and 13 years, are crown fractures without pulp exposure followed by concussions and subluxations [2–4]. Less frequently reported injuries are avulsions, extrusions, lateral luxations and crown fractures with pulp exposure. Less frequent are root fractures, intrusions and crown root fractures [4]. Combination injuries are common findings, especially crown fractures with minor luxation injuries (concussions and subluxations) [4].

A detailed history of the traumatic event together with a thorough evaluation of the injury based on a careful clinical and radiographic examination, are essential for an accurate diagnosis of the type and severity of the insult. This allows proper treatment planning and assessment of long-term prognosis [5]. Radiological examination is an essential part of the diagnostic assessment of a patient with a traumatic dental injury and initial baseline intraoral radiographs are mandatory for following up a traumatic dental injury [6]. Optimizing patient care depends also on the proper interpretation of radiological images [7]. Traumatic dental injuries, often presenting as emergency situations in dental practice, benefit from an accurate and immediate assessment [8]. When urgent care is provided in accordance with treatment strategies conform by e.g. IADT guidelines, treatment outcome will be more favourable and with lower complication rates [9]. Therefore, the development of radiodiagnostic skills is crucial for an adequate management of the dental trauma patient. Periapical radiographs are highly accessible, routinely used and recommended by the International Association of Dental Traumatology as standard for identifying the extent, type and severity of a dentoalveolar injury [10]. Generally, intraoral radiographs (2D imaging) are the first choice after dentoalveolar injury [11]. Detection of minimal tooth displacements, root fractures and alveolar bone fractures is less accurate using intraoral images as compared to Cone Beam Computed Tomography (CBCT) [12]. Lack of a third dimension and anatomical superposition limit a qualitative diagnosis [6, 13]. In more severe TDIs with bony involvement, CBCT adds useful information in order to define the complexity of the damaged structures and to reveal information hidden in other dimensions. Therefore, CBCT assessment is recommended in cases with doubtful diagnosis. The potential to provide new information not demonstrated by conventional scans allows for a more precise diagnosis, allowing a more targeted treatment plan thereby increasing outcome efficacy [11, 14]. Yet, interpreting CBCT images is more difficult than routine 2D radiodiagnosis, necessitating adequate training and a further learning process [14, 15]. As CBCT training is not always included in the regular dental curriculum and in paediatric dentistry specialist training, CBCT-reading may require more advanced education [16].

Many patients with TDIs are seen by paediatric dentists. Currently, the role and diagnostic performance of the paediatric dentist in TDI diagnosis is hardly discussed in literature. Most publications evaluate only the level of knowledge of dentists about the management of TDIs but not their (radio)diagnostic performance [17, 18]. In addition, to the best of our knowledge, there are no reports assessing the impact of the level of dental trauma management experience on (radio)diagnostic performance. Further, it has been shown that level of experience correlates well with self-assessed confidence and perception of competence, but not necessarily with actual performance [19, 20]. The latter finding might impact negatively on perceived needs in continuing professional development and thus clinical performance.

The aim of the present study is to assess the impact of experience with traumatic dental injuries (TDI) on the radiodiagnostic performance and self-assessed confidence of paediatric dentists when facing traumatic dental injuries and whether this is influenced by the imaging technique used (2D versus 3D).

## Materials and methods

Participants of this exploratory study were paediatric dentists, recruited among the members of the Belgian Academy of Paediatric Dentistry (BAPD). Participation was voluntary. A questionnaire was used to collect information regarding personal and professional profile of the participants: gender, practice of paediatric dentistry, experience with the management of dental trauma (frequency of dental trauma cases in their dental practice; number of dental trauma cases in their patient population; referral pattern) and familiarity with CBCT-imaging (training, access).

Based on the reported frequency of TDI among their patient population and the reported referral pattern in relation to TDIs, two levels of experience with TDI were distinguished. Participants reporting a low frequency of treating TDI and referring complex trauma cases were categorized as having a low level of experience with TDIs. Observers were categorized as high level experienced with TDIs when frequently (a new case at least monthly) seeing and self-managing all dental trauma cases.

Familiarity with CBCT was rated according to the level of training received and the accessibility of the imaging modality for the practitioner.

Participants were asked to assess 2D and 3D radiographic records of twenty patients having experienced a dental trauma. They were selected from the database of a single operator (G.V.G) and occurred in the time period between July 2010 and October 2016. The mean age at trauma was 8.8 years (± 2.4), with a range from 5 to 15 years, including 9 girls and 11 boys (Table 1). Out of the 35 teeth that were affected, 10 teeth presented with damage limited to the hard tissues, 9 showed lesions limited to the periodontal ligament and 16 teeth presented a combination of both. Pathological conditions included the presence of apical pathology in 12 teeth and inflammatory root resorption in 10 teeth (Table 1). For each case intra-oral radiographs and CBCT images were available, acquired within four months after dental trauma and with a maximum of three weeks between 2 and 3D imaging. A diversity of dental trauma scenarios was selected in order to provide a wide range of TDI situations. All cases were pseudonomized.

**Table 1:** Distribution and description of dental trauma cases

Dental trauma cases N = 20		
Gender		
Male	11	
Female	9	
Age (years)		
Range	5–15	
Mean (± SD)	8.8 (± 2.4)	

Traumatized teeth N = 35		
Type of injury		
Damage to hard dental tissues	10	
Damage to periodontal ligament and/or bone	9	
Combined lesions	16	
Pathological conditions		
Apical Pathology	12	
Inflammatory Root Resorption	10	

*SD* standard deviation

For 3D imaging, a 3D Accuitomo 170® CBCT (Morita, Kyoto, Japan) was used with voxel size between 0.125 mm and 0.160 mm for respectively a small (6 × 6 cm) and medium (8 × 8 cm) field of view and exposure parameters 90 kV and 5 mA. Digital images were made with a wall-mounted Dental X-ray capturing device (SIRONA Heliodent DS Intraoral Sirona Dental Systems, Bensheim, Germany) in the private practice and with a Minray® X-ray machine (Soredex, Tuusula, Finland) at the University Hospital, both with settings: 65 kV, 7 mA and exposure time of 0.08 s and with use of the paralleling long cone technique. Occlusal and periapical images were obtained using 5 × 7 cm (VistaScan® image plate, Dürr Dental AG, Bietigheim-Bissingen, Germany), respectively 3 × 4 cm phosphor plates [Digora Optime UV System (Soredex, Tusuula, Finland) or the VistaScan mini plus (Dürr Dental AG, Bietigheim-Bissingen, Germany)]. Two-dimensional images were randomly presented in a PowerPoint® (2013, Microsoft) presentation, after removing personal identifiers. CBCT data were made available as a OneVolumeViewer.exe® file (Morita, Kyoto, Japan), allowing observers to adjust and scroll through the volumes freely.

Both 2D and 3D images of the twenty dental trauma cases were presented to the participants in random order. The observations were organized in two separate sessions, with a 3-month interval, avoiding presenting both 2D and 3D images in the same session. For standardization purposes, a time limit was set with 10 min for the evaluation of a 3D record and 3 min for the evaluation of a 2D image. Sessions were organized in a computer class and were supervised by two persons. Access to supplementary material or guidelines (e.g. Dental Trauma Guide) was not possible. Each case was presented with additional and relevant information about the clinical history of the dental trauma, gender, trauma history (where, when and what happened), presence of fistula, tooth vitality, tooth mobility, complaints, first aid procedures, time lapse between imaging and dental trauma and a clinical illustration. During the first session, five training and three calibration cases, no part of the main study, were presented to the participants.

Radiodiagnostic performance was assessed at three different levels: detection, identification and interpretation of findings. Detection consists of noting that a potentially significant finding is present that merits further analysis. Identification refers to the description of the lesion and interpretation relates to the process of characterizing the lesion as being of a specific type.

**Fig. 1:**
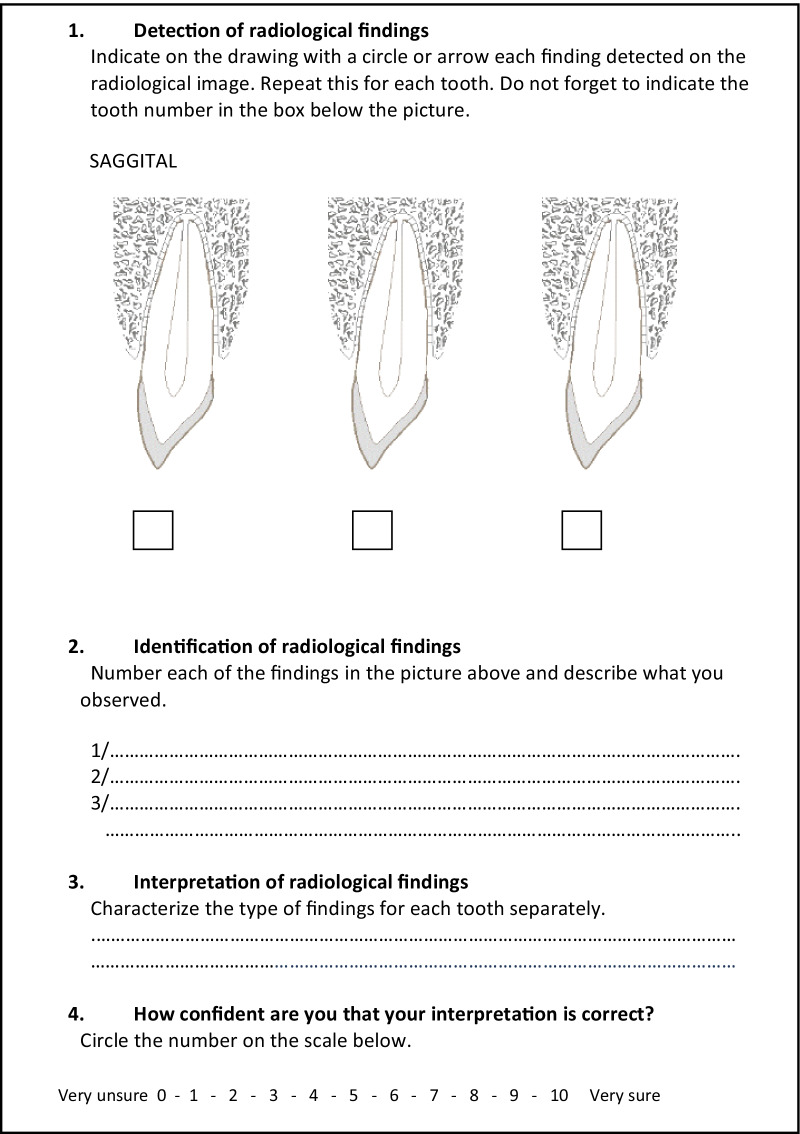
Example of a pictorial reporting sheet for sagittal dimension

Before starting the first session, observers were informed about how to complete the pictorial reporting sheet for each image (2D and 3D) by indicating each radiologic finding on a transversal schematic representation for 2D images and on axial, transversal and sagittal schematic representations for 3D images (Fig. 1). Participants were instructed to indicate with a circle or arrow the location of any potentially significant finding, allowing the assessment of their performance in detecting findings. They were then asked to identify each of the findings by describing them. Finally, they were asked to define the specific type of lesion, as a reflection of their interpretation capacity.

In addition, observers were invited to self-assess how confident they felt about their final interpretation using a visual analogue scale (VAS) from 0 to 10 (0: not confident at all to 10: very confident).

Performance of the observers was assessed by comparing their results with that of a benchmark. The standard of reference for the detection, identification and interpretation of the radiologic findings was established by two experts, a paediatric dentist with large experience in dental traumatology (exclusive practice) and a dentist specialized in oral imaging (university teacher), in consensus. The scoring of the different images was undertaken under identical circumstances.

Information obtained from the questionnaires and the reporting forms was entered into an Excel worksheet (Excel 2013 - Microsoft Office 2013) and analysed with S-plus 8.0 for Linux (Tibco, Palo Alto, CA, USA). Study population characteristics were summarized by a frequency distribution.

The scoring behavior of the observers was also explored. A multivariate space spanned by three predefined quality parameters (number of false positives, number of false negatives and number of wrong identifications) was set up for 2D and 3D images separately. Observers were defined as outliners when showing more mistakes than the median number on both 2D and 3D, when data analysis revealed a Mahalanobis distance, based on a robust estimation of covariance and location, larger than the 99.9th percentile of a chi-square distribution with 3 degrees of freedom. Outliers were removed from the group of study participants.

The impact of the level of experience with the management of dental trauma on radiodiagnostic performance was verified separately for 2D and 3D images and compared to each other. Results were analysed using a linear mixed model with imaging modality and experience as crossed fixed factors and observer and case as fixed random factors. In order to allow comparisons between both oral imaging techniques, analyses were limited to findings visible both on 2D and 3D images. Residual analysis by means of a normal quantile plot and residual dot plot showed that residuals were normally distributed with the same variance. Statistical significance level was set at 0.05.

In addition, the impact on self-assessed confidence was explored. Confidence scores were analysed using a linear mixed model with imaging modality and level of experience as crossed fixed factors and observer and case as fixed random factors. A normal quantile plot of the residuals was made to confirm that the basic assumptions of the model were met. Statistical significance level was set at 0.05.

## Results

Seventeen, out of 70 addressed paediatric dentists, agreed to participate in the study and thirteen observers completed both scoring sessions: one observer did not participate in any session without notification, two observers were not able to participate in the second session (because of practical reasons) and one observer was excluded because of a high number of missing answers. Scores of four observers were removed, based on predefined parameters, because they were considered as outliers, either for 2D or for 3D images. Data obtained from the remaining nine observers (six female and three male paediatric dentists) was included in the analyses. The personal and professional profile of these observers is shown in Table 2.

**Table 2:** Observers characteristics

Observers N = 9	
*Personal information*	
Sex	
Male	3
Female	6
*Clinical experience as paediatric dentist*	
Years of experience	
< 5 years	3
5–10 years	1
11–20 years	3
21–30 years	0
31–40 years	2
Proportion patients < 18 years	
< 10%	1
55–80%	6
100%	2
*Clinical experience with dental trauma*	
New trauma case seen at least	
Weekly	3
Monthly	3
Every 3 months	2
Every 6 months	1
Treating TDI	
All cases themselves	4
Referral of complex cases	5
TDI among patient population	
< 5	2
5–10	3
11–20	3
21–30	1
31–40	0
*Clinical experience with CBCT*	
CBCT training	
Never	6
Postgraduate training	2
Additional courses	1
Possibility of taking CBCT	
Own office	2
Referral to hospital	4
Referral to colleague	1
Never need a CBCT	2

*TDI* Traumatic Dental Injuries, *CBCT* Cone Beam Computed Tomography

When the participants were questioned regarding their experience with the management of dental trauma, three observers reported to see a new dental trauma case at least once a week, three at least monthly, two every three months and one only every six months. Four observers reported treating all dental trauma cases themselves, while the remaining five referred the more complex ones. Three observers were categorised as having a high level of experience with TDI, two of them being older paediatric dentists and all of them were treating only or mostly children. The remaining six participants were classified in the “low TDI experience” category; five of them referring complex trauma cases to more specialized centres (Table 2).

Almost all observers had a low familiarity with the CBCT technique. Six observers never received a specific training in the use of CBCT, two were trained during their postgraduate program in paediatric dentistry and one followed an additional course after graduation. Two observers had access to CBCT in their dental office, four referred to a hospital, one referred to a colleague nearby and two observers indicated that referral for CBCT was considered unnecessary and never requested a CBCT in case of dental trauma (Table 2).

The impact of level of experience with dental trauma management on radiodiagnostic performance is presented separately for detection of the finding, identification (description of type of finding), and correct interpretation of the nature of the traumatic injury. For each outcome, results were calculated according to the level of observers’ experience with TDIs and this for 2D and 3D imaging separately (Table 3). Overall, the performance of the paediatric dentists was moderate to poor with sensitivity percentages ranging between 34.8 and 56.4%. No statistically significant differences were present between low and high TDI experienced observers, for none of the outcomes and neither imaging modality. Among observers with low experience with TDI, the sensitivity for the detection and identification of findings was significantly higher on 3D than on 2D (*P* < 0.05). Obtained sensitivity scores surpassed those obtained by paediatric dentists with high level of experience, without reaching statistical significance.

**Table 3:**
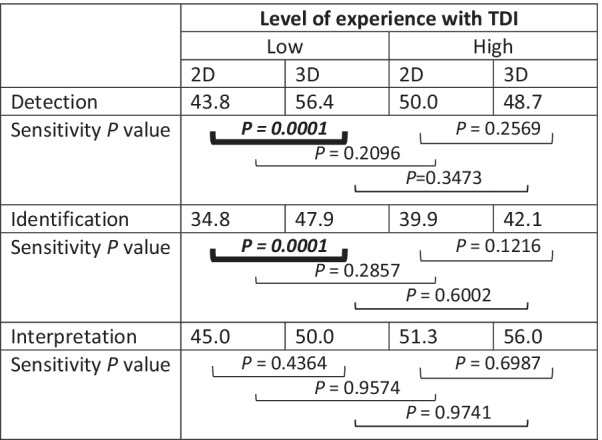
Impact of level of experience on detection, identification and interpretation on 2D and 3D images

*P* < 0.05 indicates statistically significant differences

*TDI* Traumatic Dental Injuries

In Table 4, observers’ self-assessed confidence is presented according to their level of experience with TDI management and this for 2D and 3D imaging separately. Self-assessed confidence levels were significantly higher in paediatric dentists with high TDI experience, both when using 2D (*P* < 0.05) and 3D images (*P* < 0.05).

**Table 4:**
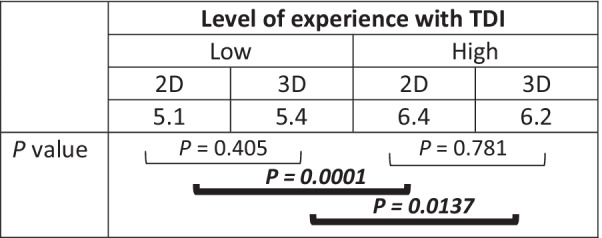
Impact of level of experience on observers’ self-assessed confidence using either 2D or 3D images

Mean confidence score as predicted by the linear mixed model

*P* < 0.05 indicates statistically significant differences

*TDI* Traumatic dental injuries

## Discussion

The objective of this study was to explore the impact of experience with dental trauma management on the detection, identification and interpretation of radiological findings by paediatric dentists, using either 2D or 3D images.

Radiodiagnostic performance is defined as the ability to detect an abnormality, identify it based on a description of image appearances and to interpret and report radiographs [21]. It is an essential part of the diagnostic process in many clinical situations, also in dental traumatology. The added value of radiographic imaging largely depends on an individual’s competence to interpret the radiograph by accurately identifying and recognizing any (ab)normality or pathology present [22].

Overall, only around half of the radiographic findings recorded by the benchmark were detected and even less of them were correctly identified. This indicates that the assessment of the radiographic images used in the present research was incomplete and suboptimal, with possible risk of overlooking items relevant for a correct diagnosis and selection of the most appropriate treatment approach. The impact of this research finding needs further exploration, but it is clear that paediatric dentists could benefit from more training in the assessment and interpretation of radiographic images, both 2D and 3D. Since the performance of the observers might also be influenced by the magnitude of the alterations induced by the traumatic event, reflecting the extent of the injuries, the impact of case complexity needs to be explored in more detail.

The use of 3D imaging improved detection as well as correct identification of findings only in dentists with low TDI experience, without evidence of an impact on correct interpretation. This finding suggests that 3D imaging cannot compensate for lack of experience. Further, this exploratory study did not yield evidence of better performance of paediatric dentists with high level of experience when using 3D images. This is in accordance with earlier findings emphasizing the need for training to adequately evaluate CBCT scans [23]. When training and experience in reading and interpreting of CBCT images is lacking, delegation of the interpretation of images to an appropriately trained dento-maxillofacial radiologist should be envisaged [24].

The fact that no proof of impact of experience level could be demonstrated might be based on differences in education and training among participants. The more experienced paediatric dentists, mostly also the older observers, did not get training in CBCT interpretation in their under- or postgraduate program contrary to younger graduates who were exposed to the use of this diagnostic modality during their education [25]. Furthermore, despite training in radiographic interpretation and diagnosis evolved considerably over the past years, leading to improved performance even regarding 2D images, educational experience in CBCT technology and interpretation is not yet as widespread. It has been demonstrated that being actively involved in CBCT training results in an increased performance in CBCT image interpretation [26]. Further, dental practitioners with more CBCT knowledge refer more frequently for CBCT, when the indication is justified [27]. The importance of the justification for CBCT paediatric examinations should be emphasized. When doubtful diagnosis after a traumatic tooth injury would justify a CBCT, an optimised patient-specific paediatric protocol should be considered as children are more radiosensitive than adults, while CBCT doses exceed those of intraoral radiographs with a factor 20 to 200 [26–31].

Considering the growing importance of CBCT in many specialties, education and training seem to be essential keys to increase diagnostic performance when referring, reading and interpreting CBCT scans [26, 32, 33]. The role and diagnostic performance of paediatric dentists in TDI diagnosis is hardly described in literature whereas the evaluation of the level of knowledge of dentists about the management of TDIs has been extensively discussed [17, 18]. Since providing dental care to dental trauma patients is an important competence within the field of paediatric dentistry, exploration of performance and its contributing factors is important.

Interestingly, self-assessed confidence levels regarding the final interpretation of the radiological findings by the high TDI experienced observers were not related to their actual performance. Experience improves self-assessed confidence, but it is has been described in the literature that competence (task performance) and self-belief (confidence) in the ability to undertake particular tasks are different concepts [34, 35]. Despite the significant correlation between TDI experience and self-assessed confidence, both on 2D and 3D, the literature demonstrates that neither clinical experience nor level of confidence have a predictive value in performance assessments [19, 20, 36]. Most of the dentists with high experience in TDI had an older age, more years of practice and were more familiar with the interpretation of 2D rather than 3D imaging techniques [37]. It could be that older clinicians used their past clinical experience to make decisions and limited their diagnosis to 2D imaging without making extra time and effort for fully interpreting 3D images, resulting in failure to recognize a normal biologic variant or to search for more anomalies [38]. On the other hand, this could have implications for clinical practice since deficiencies in self-assessment skills can lead to overconfidence, contributing to diagnostic error, a situation that might present risks for patient safety [39].

The statement *‘one can only see what one knows or the eyes look, but the brain sees’* by JW von Goethe in 1819, underlines the importance of obtaining feedback and follow-up. Also, a number of subjective elements, such as an individual’s attitude, interest, commitment and practice consistency, are factors to consider; they could explain the disparity between radiological diagnostic performance and professional seniority [40]. It is well known that people are not always accurate in estimating their performance and in recognizing their own incompetence [41, 42], also in the medical field [43]. Nevertheless, the assessment of one’s competence could assist in identifying the training and learning needs in continuing professional development [34, 44]. It is clear that additional training in both the theoretical and the practical aspects of CBCT is important for an optimal and safe use of CBCT in the dentoalveolar region [45]. There might be a need to update training in radiodiagnosis in the undergraduate dental curriculum, both using 2D and 3D imaging, in relation to specific clinical indications such as dental trauma. This is certainly the case also for general dental practitioners since they will be confronted with dental trauma cases needing urgent decision making during on call services.

The study described in this report has several limitations. The most important one is the low number of participating paediatric dentists. Higher numbers of observers could have increased statistical power and representativeness of the results. In addition, this would have enriched the cognitive abilities measurements and enhanced the clinical translation of the results of this study.

Another limitation of the present study, possibly influencing the performance of the participants, is the fact that the information was not presented as in a clinical setting. Findings were retrieved from patient records and periapical radiographs were presented to the participants, incorporated in a power point presentation, not allowing the patient’s clinical examination and without the possibility of manipulating the periapical radiographs. The latter was not the case for the CBCT data where the observers were able to view the entire CBCT volume, using image enhancement tools as zoom, brightness and contrast. Further, because of the overall low familiarity with CBCT, its impact on diagnostic performance could not be explored.

This study cannot yield conclusive results and should therefore be considered as exploratory. Its results show that further research in this field is needed. An interesting follow-up to this study could be what treatment the dentists would propose for each specific trauma case, based on either imaging modality, and explore the possible impact on treatment outcome and prognosis.

## Conclusion

In this exploratory study, observers’ experience with the management of traumatic dental injuries was not in accordance with their ability to detect, recognize and interpret correctly radiological findings in case of a traumatic event, neither on 2D or on 3D. This indicates that the benefits of using CBCT as a more accurate diagnostic tool in TDI demands more CBCT-related training in order to cover knowledge gaps. Alternatively, image interpretation could be delegated to an appropriately trained dento-maxillofacial radiologist. Further, it was confirmed that self-assessed confidence was related to the level of experience, without demonstrating an impact of treatment modality used.

## Data Availability

The datasets generated and/or analyzed during the current study are not publicly available due to privacy policies of the University Hospitals Leuven but are available from the corresponding author on reasonable request.

## Abbreviations

TDI: Traumatic dental injuries
CBCT: Cone beam computed tomography

## References

[1] Petti S, Glendor U, Andersson L (2018). World traumatic dental injury prevalence and incidence, a meta-analysis-One billion living people have had traumatic dental injuries. Dent Traumatol.

[2] Ivancic Jokic N, Bakarcic D, Fugosic V, Majstorovic M, Skrinjaric I (2009). Dental trauma in children and young adults visiting a University Dental Clinic. Dent Traumatol.

[3] Díaz JA, Bustos L, Brandt AC, Fernández BE (2010). Dental injuries among children and adolescents aged 1–15 years attending to public hospital in Temuco. Chile Dent Traumatol.

[4] Lauridsen E, Hermann NV, Gerds TA, Kreiborg S, Andreasen JO (2012). Pattern of traumatic dental injuries in the permanent dentition among children, adolescents, and adults. Dent Traumatol.

[5] Andreasen F, Andreasen J, Tsukiboshi M (2007). Examination and diagnosis of dental injuries, in Textbook and Color Atlas of Traumatic Injuries to The Teeth.

[6] Cohenca N, Simon J, Roges R, Morag Y, Malfaz J (2007). Clinical indications for digital imaging in dento-alveolar trauma. Part 1: traumatic injuries. Dent Traumatol..

[7] Myers GL (2019). Evaluation and diagnosis of the traumatized dentition. Dent Traumatol.

[8] Moule A, Cohenca N (2016). Emergency assessment and treatment planning for traumatic dental injuries. Aust Dent J.

[9] Bücher K, Neumann C, Thiering E, Hickel R, Kühnisch J (2013). International Association of Dental Traumatology. Complications and survival rates of teeth after dental trauma over a 5-year period. Clin Oral Investig..

[10] Bourguignon C, Cohenca N, Lauridsen E, Flores MT, O'Connell AC, Day PF, Tsilingaridis G, Abbott PV, Fouad AF, Hicks L, Andreasen JO, Cehreli ZC, Harlamb S, Kahler B, Oginni A, Semper M, Levin L (2020). International Association of Dental Traumatology guidelines for the management of traumatic dental injuries: 1. Fract luxations Dent Traumatol.

[11] Kullman L, Al SM (2012). Guidelines for dental radiography immediately after a dento-alveolar trauma, a systematic literature review. Dent Traumatol.

[12] Cohenca N, Shemesh H (2015). Clinical applications of cone beam computed tomography in endodontics: a comprehensive review. Quintessence Int.

[13] Fayad M, Johnson B (2016). 3D imaging in endodontics: a new era in diagnosis and treatment.

[14] Cohenca N, Silberman A (2017). Contemporary imaging for the diagnosis and treatment of traumatic dental injuries: a review. Dent Traumatol.

[15] Scarfe WC, Li Z, Aboelmaaty W, Scott SA, Farman AG (2012). Maxillofacial cone beam computed tomography: essence, elements and steps to interpretation. Aust Dent J.

[16] Oenning A, Jacobs R, Pauwels R, Stratis A, Hedesiu M, Salmon B, DIMITRA Research Group (2018). Cone-beam CT in paediatric dentistry: DIMITRA project position statement. Pediatr Radiol..

[17] Hartmann RC, Rossetti BR, Siqueira Pinheiro L, de Figueiredo JAP, Rossi-Fedele G, Gomes S, de Borba MG (2019). Dentists' knowledge of dental trauma based on the International Association of Dental Traumatology guidelines: a survey in South Brazil. Dent Traumatol..

[18] Krastl G, Filippi A, Weiger R (2009). German general dentists' knowledge of dental trauma. Dent Traumatol.

[19] Gilmour AS, Welply A, Cowpe JG, Bullock AD, Jones RJ (2016). The undergraduate preparation of dentists: confidence levels of final year dental students at the School of Dentistry in Cardiff. Br Dent J.

[20] O'Donoghue D, Davison G, Hanna LJ, McNaughten B, Stevenson M, Thompson A (2018). Calibration of confidence and assessed clinical skills competence in undergraduate paediatric OSCE scenarios: a mixed methods study. BMC Med Educ.

[21] Piper KJ, Paterson A (2009). Initial image interpretation of appendicular skeletal radiographs: a comparison between nurses and radiographers. Radiography.

[22] Hardy M, Barrett C (2003). Interpreting trauma radiographs. J Adv Nurs.

[23] Ahmad M, Jenny J, Downie M (2012). Application of cone beam computed tomography in oral and maxillofacial surgery. Aust Dent J.

[24] Patel S, Harvey S (2021). Guidelines for reporting on CBCT scans. Int Endod J.

[25] Giray FE, Peker S, Yalcinkaya SE, Kargul B, Aps J (2019). Attitudes and knowledge of paediatric dentists' on digital radiography and cone beam computed tomography. J Pak Med Assoc.

[26] Parashar V, Whaites E, Monsour P, Chaudhry J, Geist JR (2012). Cone beam computed tomography in dental education: a survey of US, UK, and Australian dental schools. J Dent Educ.

[27] Zain-Alabdeen EH, El Khateeb SM (2018). Comparison of knowledge and perspectives toward cone-beam computed tomography among dentists in three Middle East regions: a cross-sectional study. S J Oral Sci.

[28] Theodorakou C, Walker A, Horner K, Pauwels R, Bogaerts R, Jacobs R, SEDENTEXCT Project Consortium (2012). Estimation of paediatric organ and effective doses from dental cone beam CT using anthropomorphic phantoms. Br J Radiol..

[29] Hedesiu M, Marcu M, Salmon B, Pauwels R, Oenning AC, Almasan O, Roman R, Baciut M, Jacobs R, DIMITRA Research Group (2018). Irradiation provided by dental radiological procedures in a pediatric population. Eur J Radiol..

[30] Oenning AC, Pauwels R, Stratis A, De Faria Vasconcelos K, Tijskens E, De Grauwe A, Jacobs R, Salmon B, Dimitra Research Group (2019). Halve the dose while maintaining image quality in paediatric Cone Beam CT. Sci Rep..

[31] Marcu M, Hedesiu M, Salmon B, Pauwels R, Stratis A, Oenning ACC, Cohen ME, Jacobs R, Baciut M, Roman R, Dinu C, Rotaru H, Barbur I, Dimitra Research Group (2018). Estimation of the radiation dose for pediatric CBCT indications: a prospective study on ProMax3D. Int J Paediatr Dent..

[32] Rabiee H, McDonald NJ, Jacobs R, Aminlari A, Inglehart MR (2018). Endodontics Program Directors’, Residents’, and Endodontists’ considerations about CBCT-Related Graduate Education. J Dent Educ..

[33] Yeung AWK, Tanaka R, Jacobs R, Bornstein MM (2020). Awareness and practice of 2D and 3D diagnostic imaging among dentists in Hong Kong. Br Dent J.

[34] Stewart J, O'Halloran C, Barton JR, Singleton SJ, Harrigan P, Spencer J (2000). Clarifying the concepts of confidence and competence to produce appropriate self-evaluation measurement scales. Med Educ.

[35] Barnsley L, Lyon PM, Ralston SJ, Hibbert EJ, Cunningham I, Gordon FC, Field MJ (2004). Clinical skills in junior medical officers: a comparison of self-reported confidence and observed competence. Med Educ.

[36] Morgan PJ, Cleave-Hogg D (2002). Comparison between medical students' experience, confidence and competence. Med Educ.

[37] Tewary S, Luzzo J, Hartwell G (2011). Endodontic radiography: Who is reading the digital radiograph?. J Endod..

[38] Bussaneli DG, Boldieri T, Diniz MB, Rivera LM, Santos-Pinto L, Cordeiro RC (2015). Influence of professional experience on detection and treatment decision of occlusal caries lesions in primary teeth. Int J Paediatr Dent.

[39] Berner ES, Graber ML (2008). Overconfidence as a cause of diagnostic error in medicine. Am J Med.

[40] Akadiri O, Olusanya A, Udeabor S, Agi C (2012). Identification and interpretation of maxillofacial plain radiographs by junior dental trainees. J West Afr Coll Surg.

[41] Kruger J, Dunning D (1999). Unskilled and unaware of it: How difficulties in recognizing one's own incompetence lead to inflated self-assessments. J Pers Soc Psychol..

[42] Dunning D, Kerri Johnson K, Ehrlinger J, Kruger J (2003). Why people fail to recognize their own incompetence. Curr Dir Psychol Sci.

[43] Rezaiefar P, Forse K, Burns JK, Johnston S, Muggah E, Kendall C, Archibald D (2019). Does general experience affect self-assessment?. Clin Teach..

[44] Colthart I, Bagnall G, Evans A, Allbutt H, Haig A, Illing J, McKinstry B (2008). The effectiveness of self-assessment on the identification of learner needs, learner activity, and impact on clinical practice: BEME Guide no. 10. Med Teach..

[45] Brown J, Jacobs R, Levring Jaghagen E, Lindh C, Baksi G, Schulze D (2014). Basic training requirements for the use of dental CBCT by dentists: a position paper prepared by the European Academy of DentoMaxilloFacial Radiology. Dentomaxillofac Radiol.

